# MRISCs protect colonic stem cells from inflammatory damage

**DOI:** 10.1186/s13619-021-00086-4

**Published:** 2021-06-03

**Authors:** Guoli Zhu, Rongwen Xi

**Affiliations:** 1 National Institute of Biological Sciences, No. 7 Science Park Road, Zhongguancun Life Science Park, Beijing, 102206 China; 2 Tsinghua Institute of Multidisciplinary Biomedical Research, Tsinghua University, Beijing, China

## Abstract

Increasing evidence suggest functional roles of subepithelial mesenchymal niche cells in maintaining intestinal stem cells and in modulating the pathogenesis of various intestinal diseases in mammals. A recent study reported the discovery of a new population of stromal cells in mice termed MAP3K2-Regulated Intestinal Stromal Cells (MRISCs); these cells reside at the base of colonic crypt and function to protect colonic stem cells during colonic inflammation by expressing the Wnt agonist R-spondin1 (Rspo1).

## Main Text

**Fig. 1:**
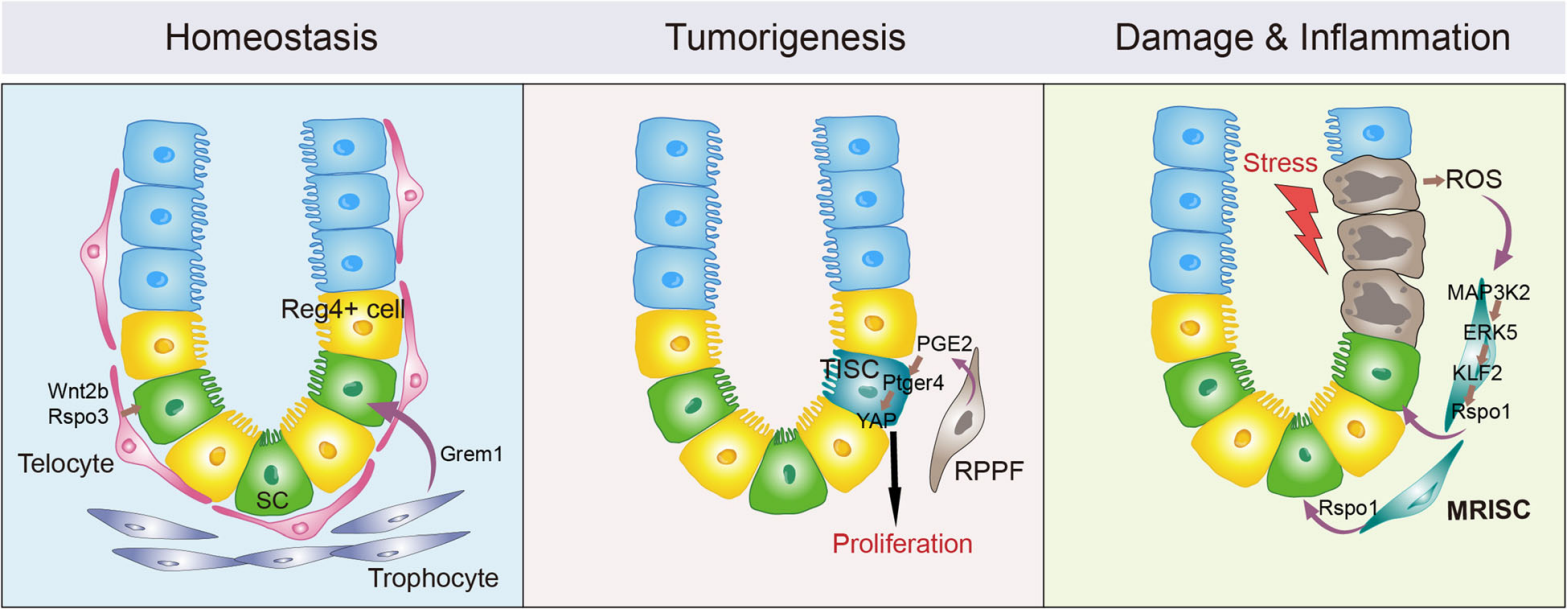
The mesenchymal niche populations at the peri-crypt regions of the normal and diseased colon in mice. Left panel. During normal homeostasis, there are three main cellular sources of niche signals for colonic stem cells (SC): the Reg4^+^ deep crypt secretory cells from the epithelial compartment, and the telocyte and trophocyte populations from the mesenchyme compartment. Middle panel. During AOM-induced tumorigenesis, a rare population of peri-cryptal PTGS2-expressing fibroblast (RPPF) cells appears and communicates directly with the adjacent tumor-initiating stem cell (TISC) via a PGE2-PTGER4-YAP signaling axis to promote TISC proliferation and tumorigenesis. Right panel. In DSS-treated colon, a stromal cell population termed as MRISC appears at the bottom of the crypt base that produces Rspo1 via a ROS-MAP3K2-ERK5-KLF2-Rspo1 signaling cascade to promote self-renewal of colonic stem cells, thereby exerting a protective effect against inflammation-induced colonic damage

The epithelium that lines the mammalian intestinal and colonic tube is continuously renewed with the progenies of stem cells that reside at the bottom of an invaginated structure termed the crypt. Multiple studies have demonstrated essential roles for a variety of peri-crypt stromal cell subpopulations in the regulation of stem cell self-renewal in the small intestine and colon (McCarthy et al. 2020a; Zhu et al. 2021). Foxl1^+^ telocytes, which form a layer meshwork positioned immediately beneath the intestinal and colonic epithelium, are essential for the maintenance of intestinal and colonic stem cells: Foxl1^+^ telocytes produce Wnt self-renewal signals (Degirmenci et al. 2018; Shoshkes-Carmel et al. 2018). In the small intestine, CD81^+^ trophocytes are positioned beneath telocytes in the crypt base. CD81^+^ trophocytes secret the BMP inhibitory signal Grem1, thereby facilitating establishment of a BMP activity gradient along the crypt-villus axis in the intestinal epithelium; this gradient specifies self-renewal vs. differentiation of intestinal stem cells (McCarthy et al. 2020b) (Fig. 1, left panel).

It is also known that stromal cells in the subepithelial compartment can be remodeled in disease and these remodeled stromal cells may in turn have an impact on the development of the disease (Kinchen et al. 2018; Roulis et al. 2020; Smillie et al. 2019). For example, a study which used APC^min^- and azoxymethane (AOM)-based intestinal tumor models revealed the emergence of a peri-cryptal prostaglandin endoperoxide synthase 2 (PTGS2)-expressing fibroblast subpopulation that contributes to the proliferation of tumor-initiating stem cells (TISCs), thereby promoting intestinal tumorigenesis (Roulis et al. 2020). Notably, this proliferation-promoting activity is driven by paracrine crosstalk involving prostaglandin E2 (PGE2)-PGE2 receptor 4 (PTGER4)-Yes-associated protein (YAP) signaling (Fig. 1, middle panel).

Wu et al. (2021) recently reported discovery of a new population of disease-associated stromal cells in a mouse model of colitis. These cells are responsive to inflammation and release the Wnt agonist Rspo1 to enhance the self-renewal of colonic stem cells, thereby promoting repair of the colonic epithelium (Wu et al. 2021) (Fig. 1, right panel). That study began with a phenotypic analysis of mice deficient for Map3k2, a serine/threonine kinase belonging to the mitogen-activated protein kinase kinase kinase (MAP3K) superfamily that has been implicated in cellular stress responses. They observed that Map3k2^−/−^ mice exhibited an enhanced colitis phenotype, with a significantly reduced number of colonic stem cells present following exposure to dextran sulfate sodium (DSS). This reduction in colonic stem cell number was accompanied by reduced expression of the Wnt agonist Rspo1.

The authors went further to determine whether Map3k2 exerts its functions in epithelial cells or stromal cells. Vil1-cre; Map3k2^fl/fl^ mice—wherein Map3k2 is specifically ablated in epithelial cells—developed a similar degree of DSS-induced colitis as control mice. In contrast, the Col1a2-cre^ERT2^; Map3k2^fl/fl^ mice (Map3k2 is specifically ablated in the subepithelial stromal cells) developed a severe colitis phenotype similar to the Map3k2^−/−^ mice. Therefore, the observed protective effect (i.e., antagonizing of DSS-induced colitis) results from Map3k2 in stromal cells.

The authors then conducted single cell analysis of the subepithelial compartment in control and colitis model mice and revealed a population of stromal cells that expresses Rspo1. These cells can be separated from other populations by a combination of several surface markers, including CD90^+^CD81^+^CD34^+^ and CD138^−^. The DSS model induction led to a marked upregulation of Rspo1 expression in these cells in wild-type but not Map3k2^−/−^ mice. Accordingly, these cells were proposed as a possible cellular source of Map3k2-regulated Rspodin1 and were termed “MAP3K2-Regulated Intestinal Stromal Cells” (MRISCs). Immunostaining revealed that MRISCs reside close to the colonic crypt base. Additionally, it is notable that scRNA-seq and ATAC-seq analyses revealed that MRISCs appear to be epigenetically and transcriptionally distinct from other subpopulations of intestinal stromal cells, including telocytes, trophocytes, and Ptgs2-expressing fibroblasts (Degirmenci et al. 2018; McCarthy et al. 2020b; Roulis et al. 2020; Shoshkes-Carmel et al. 2018).

MRISCs from DSS-treated wild-type and Map3k2^−/−^ colons were then sorted and examined in a co-culture system with colonic organoids. The authors observed that both the wild-type and the Map3k2^−/−^ MRISCs showed similar organoid growth-promoting activities. However, MRISCs derived from wild-type but not Map3k2^−/−^ mutant mice exhibited enhanced growth-promoting activities. It was also noted that the organoid growth-stimulating activity of MRISCs was blocked by an Rspo1 neutralizing antibody. Further, organoid growth-stimulation was observed upon the addition of recombinant Rspo1 to organoids cultured without MRISCs. The authors also performed in vivo rescue experiments wherein MRISCs were surgically transplanted to the colonic lamina: the wild-type MRISCs had a significantly stronger ability to alleviate epithelial damage compared to Map3k2^−/−^ MRISCs. Thus, MRISCs exert protective effects against inflammation-induced colonic damage by promoting colonic stem cell activity via Rspo1, and this function requires Map3k2.

To understand how Rspo1 is regulated by Map3K2 in MRISCs, the authors combined multiple approaches, including gene expression analysis, chromatin binding analysis of transcription factors as well as functional analyses in MRISCs, and eventually revealed a signaling cascade that regulates the expression of Rspo1 in MRISCs: tissue damage induces reactive oxygen species (ROS), which causes the activation of MAP3K2 and consequently the activation of ERK5 and the transcription factor KLF5, which then promotes the transcription of Rspo1. Thus, MRISCs protect colon against damage by providing Rspo1 via a ROS-MAP3K2-ERK5-KLF2 signaling cascade (Fig. 1, right panel).

The work done by Wu and colleagues expands our view of niche regulation of intestinal stem cells by revealing a new population of mesenchymal cell niche that exerts a protective function to colonic stem cells during colonic inflammation. The study also raises several open and exciting questions. It is of great interest to study the potential roles of this identified pathway in human diseases, particularly inflammatory bowel diseases and colitis-associated colorectal cancer. As MAP3K2 is expressed in many other stromal cell populations, does the identified signaling cascade also function in other stromal cells to regulate Rspo expression? Finally, due to extensive heterogeneity and dynamic nature associated with the mesenchymal cell populations, one significant obstacle in intestinal mesenchymal cell research is the lack of consensus on the exact identity and marker expression of each sub-populations of intestinal mesenchymal cells. As for the MRISC population described in the study, these cells also express CD81 and other signature genes found in the trophocyte population in mouse small intestine. As a comparative analysis of the scRNA-seq data revealed substantial similarities among mesenchymal cell populations in the small intestine and colon (McCarthy et al. 2020a), it would be interesting to determine whether the MRISCs belong to or are significantly overlapped with the trophocyte population in colon. The Wu et al. (2021) study reinforces the idea that remodeling of the mesenchymal cell niche can significantly influence the activity of intestinal stem cells and the development of intestinal diseases. In addition, the identification and characterization of a signaling cascade in the regulation of Rspo1 expression, a major intestinal stem cell niche signal, also opens new potential avenues for therapeutic intervention of intestinal inflammatory diseases and cancer.

## Abbreviations

MRISCs: MAP3K2-Regulated Intestinal Stromal Cells
Rspo1: R-spondin1
PTGS2: prostaglandin endoperoxide synthase 2
TISCs: tumor-initiating stem cells

## References

[CR1] Degirmenci B, Valenta T, Dimitrieva S, Hausmann G, Basler K (2018). GLI1-expressing mesenchymal cells form the essential Wnt-secreting niche for colon stem cells. Nature.

[CR2] Kinchen J, Chen HH, Parikh K, Antanaviciute A, Jagielowicz M, Fawkner-Corbett D, Ashley N, Cubitt L, Mellado-Gomez E, Attar M (2018). Structural remodeling of the human colonic mesenchyme in inflammatory bowel disease. Cell.

[CR3] McCarthy N, Kraiczy J, Shivdasani RA (2020). Cellular and molecular architecture of the intestinal stem cell niche. Nat Cell Biol.

[CR4] McCarthy N, Manieri E, Storm EE, Saadatpour A, Luoma AM, Kapoor VN, Madha S, Gaynor LT, Cox C, Keerthivasan S (2020). Distinct mesenchymal cell populations generate the essential intestinal BMP signaling gradient. Cell Stem Cell.

[CR5] Roulis M, Kaklamanos A, Schernthanner M, Bielecki P, Zhao J, Kaffe E, Frommelt L-S, Qu R, Knapp MS, Henriques A (2020). Paracrine orchestration of intestinal tumorigenesis by a mesenchymal niche. Nature.

[CR6] Shoshkes-Carmel M, Wang YJ, Wangensteen KJ, Tóth B, Kondo A, Massassa EE, Itzkovitz S, Kaestner KH (2018). Subepithelial telocytes are an important source of Wnts that supports intestinal crypts. Nature.

[CR7] Smillie CS, Biton M, Ordovas-Montanes J, Sullivan KM, Burgin G, Graham DB, Herbst RH, Rogel N, Slyper M, Waldman J (2019). Intra-and inter-cellular rewiring of the human colon during ulcerative colitis. Cell.

[CR8] Wu N, Sun H, Zhao X, Zhang Y, Tan J, Qi Y, Wang Q, Ng M, Liu Z, He L, Niu X, Chen L, Liu Z, Li HB, Zeng YA, Roulis M, Liu D, Cheng J, Zhou B, Ng LG, Zou D, Ye Y, Flavell RA, Ginhoux F, Su B (2021). MAP3K2-regulated intestinal stromal cells define a distinct stem cell niche. Nature.

[CR9] Zhu G, Hu J, Xi R (2021). The cellular niche for intestinal stem cells: a team effort. Cell Regen.

